# Suicide rates in Germany in lockdown and non-lockdown phases during the COVID-19 pandemic in 2020/2021

**DOI:** 10.1371/journal.pone.0331784

**Published:** 2025-09-08

**Authors:** Anne Elsner, Roland Mergl, Antje-Kathrin Allgaier, Ulrich Hegerl

**Affiliations:** 1 German Foundation for Depression and Suicide Prevention, Leipzig, Germany; 2 University of the Bundeswehr Munich, Institute of Psychology, Neubiberg, Germany; 3 Department of Psychiatry, Psychosomatic Medicine and Psychotherapy, University Hospital Frankfurt, Goethe University Frankfurt (Goethe Research Professorship), Frankfurt am Main, Germany; Facultad Latinoamericana de Ciencias Sociales Mexico, MEXICO

## Abstract

Contrary to the cautions of many, suicide rates during the first COVID-19 pandemic lockdown in Germany did not rise, but declined. With the accessibility of the 2021 weekly suicide data, it was possible to analyze suicide rates and the proportion of suicide methods during the lockdown and non-lockdown phases and during the first 21 months of the pandemic compared to the previous ten years. An interrupted time-series analysis based on a linear regression was used. For the comparisons of predicted and observed suicide rates, excess suicide mortality rates (ESMR) were chosen among others. Changes in the choice of method were analyzed by comparing the rates of different methods in lockdown- and non-lockdown phases. Since the start of the COVID-19 pandemic, suicide mortality in 2020/2021 was significantly higher than expected (ESMR = 1.0161; 95% CI: 1.0005; 1.0317). Registered suicide mortality was lower than expected during the lockdown phases in these years (ESMR = 0.9477; 95% CI: 0.9128; 0.9825) and higher during non-lockdown phases (ESMR = 1.0353; 95% CI: 1.0178; 1.0528). A MANOVA revealed a significant difference of the absolute number of suicides between lockdown and non-lockdown periods (factor “lockdown”: F(1,100) = 9.47; p = 0.003). A shift of suicide methods in the comparison of these periods could not be detected. Our results are in line with the rise in yearly absolute numbers of suicides in 2022 in Germany and illustrate that even though suicide rates declined during lockdowns, there was a general rise since the beginning of the COVID-19 pandemic, especially during the non-lockdown periods. This hints towards a “postponement effect”, probably as loosening of the lockdown restrictions makes suicide methods more easily accessible and more suicides than expected are present in the following non-lockdown periods. More research is needed to better understand the mechanisms influencing suicidality during lockdowns and the COVID-19 pandemic in general.

## Introduction

With the start of the COVID-19 pandemic, practitioners and researchers cautioned about a negative impact of the disease and its counter-measures on suicide mortality [1,2]. Shortly after, it was shown that the prevalence of depression, anxiety, insomnia and PTSD among populations affected by COVID-19 was higher than in the general population [3]. Also, daily COVID-19 infection rates and reductions in human mobility were associated with increased prevalence of depression and anxiety [4].

People’s lives have been altered by the lockdowns concerning daily aspects such as work, leisure time and social interaction in general. Furthermore, lockdowns have the potential to aggravate mental illnesses [3,5] or even suicidality, the latter due to social distancing and lesser consultation of therapists [4]. On the other hand, lockdowns might have a suicide preventive effect [6]. This might be due to factors like strengthened social control leading to a better supervision of individuals with increased suicidality, less access to suicide methods with high lethality like railway suicides as well as less opportunities for suicides in homes [6]. When comparing suicide numbers in Germany during the first nation-wide lockdown (03/22/2020-05/06/2020) to a ten-year baseline, it was shown that suicide mortality was significantly lower than expected [6], which was in line with previous international studies [7,8]. In the period between the first and the second lockdown (05/07/2020-12/15/2020), the registered suicide mortality was numerically, but not significantly higher than expected [6]. At the time of the analysis, weekly suicide rates were only available for the year 2020 and it remained open how the suicide mortality would develop during the second lockdown in Germany (12/16/2020-02/28/2021).

One explanation to consider for the different suicide rates in lockdown vs. non-lockdown phases could be the use of different suicide methods, since they vary in lethality [9,10]. In order to find out whether the measures taken against the pandemic had an influence on the choice of suicide methods, a possible shift of suicide methods in lockdown phases could be investigated. In our first study, we could detect only a partial shift in suicide methods during the first lockdown compared to the ten years before, which showed a significant increase of the percentage of the suicide method categories jumping and ‘other methods’ and a decrease of poisoning and lying in front of a moving object [6]. The interpretation of these findings was difficult, especially linking them to the measures taken against the pandemic, since information about the location of the suicide could not be obtained by the Research Data Centre for reasons of confidentiality.

In the present study, we analyzed weekly suicide data from the years 2020 and 2021. This made it possible to investigate the dynamics of suicide mortality in the timespan of the first and the second lockdown and the non-lockdown phases of these years. Taking together suicide data for two lockdowns and two non-lockdown time-spans allowed for a greater statistical power, so as to find possible significant differences of suicide mortality and changes in choices of suicide methods during these intervals compared to a ten-year baseline. Suicide data were analyzed using descriptive and exploratory statistics concerning the following research questions:

1. Do suicide rates in Germany during the COVID-19 Pandemic in the years 2020 (weeks 13–52) and 2021 (weeks 1–52) differ significantly from the rates of the previous ten years?
2. Do suicide rates in Germany during the first and second lockdown (03/22/2020-05/06/2020 and 12/16/2020-02/28/2021 taken together) differ significantly from the rates of the previous ten years?
3. Do suicide rates in Germany during the non-lockdown phases in 2020/2021 (05/07/2020-12/15/2020 and 03/01/2021-12/31/2021 taken together) differ significantly from the rates of the previous ten years?
4. Do suicide rates in Germany during the first and second lockdown (03/22/2020-05/06/2020 and 12/16/2020-02/28/2021 taken together) differ significantly from the rates during the non-lockdown phases in 2020/2021 (05/07/2020-12/15/2020 and 03/01/2021-12/31/2021 taken together)?
5. Was there a shift in suicide methods during the first and second lockdown (03/22/2020-05/06/2020 and 12/16/2020-02/28/2021 taken together) compared to the non-lockdown phases in 2020/2021 (05/07/2020-12/15/2020 and 03/01/2021-12/31/2021 taken together)?

## Methods

### Data collection

The weekly absolute number of suicides (ICD-10 codes: X60-X84) by age group as well as gender for the period 2010–2021 was extracted from the Causes of Death Statistics provided by the Research Data Centre of the Statistical Offices of the Federal States in Germany. These data were recorded officially and had been fully anonymized. Therefore, we waived a review by an ethics committee. Also, participant consent was not obtained since the data was fully anonymized. The data of the Research Data Centre can be applied by research institutions in exchange for user fees. Population data (stratified by both gender and age groups) were derived from a publicly accessible database (genesis) from the Federal Statistical Office of Germany [11].

### Methods used for suicide

Within the Causes of Deaths Statistics, methods of suicidal actions were given according to ICD-10 codes X60-X84. In the context of our study, methods were categorized as follows: self-poisoning (X60-X69), hanging (X70), drowning (X71), firearms (X72-X75), cutting by sharp objects (X78), jumping from high places (X80), lying in front of a moving object (X81, X82), and other suicide methods (X76, X77, X79, X83, X84).

### Definition of lockdown intervals

As Germany consists of 16 federal states with autonomous lockdown regulations, we defined lockdown intervals with the highest national overlap [6]. The first lockdown began on March 22 of 2020 and ended on May 6 of the same year; in this period there were Germany wide contact restrictions as well as regulations for social distancing, followed by less strong regulations in most areas of everyday life [6]. The second so-called “hard lockdown” was defined to start on December 16 in 2020 (as opposed to the “lockdown light” which started on November 2 in 2020) and lasted until February 28 in 2021.

### Statistical analysis

In order to give an answer to the question of whether suicide rates during the first and second lockdown were significantly different from the expected suicide rates as predicted from the same period during a 10-year baseline, an interrupted time-series analysis was chosen. For this purpose, two subperiods of the second lockdown (01/01–02/28 and 12/16–12/31; years: 2010–2019) were defined. Suicide data from each subperiod of the second lockdown period (e.g., 12/16–12/31; years: 2010–2019) were selected to compute a corresponding predictor (e.g., 12/16–12/31/2020); next, predictors were compared with registered suicide data of this subperiod. Thus, a linear regression analysis was computed with the yearly suicide rate during the baseline period (2010–2019) being the dependent variable and “year” being the independent variable. Based on this model, predictors were given for the two subperiods of the second lockdown period (see above). The result of this analysis was an expected suicide rate (ESR) following the formula: ESR = (expected number of suicides (E)/ population in the first or second subperiod of the second lockdown period in 2020/2021) * 100.000). In this context, the population figures used for the calculation of ESR were derived from end-period registry counts (deadline: day 12/31 of the corresponding year) [11]. Based on the latter formula, E was computed. Next, E and ESR for the first and second subperiod of the second lockdown period were added up to get E and ESR for the total second lockdown period. In a next step, the corresponding data for the first and second lockdown were merged.

Then, the excess suicide mortality rate (ESMR) was calculated being the quotient of the observed number of suicides (O) and E (ESMR = O/E) [6]. The 95% confidence interval (CI) of the ESMR was calculated, too (for details see [6]). The number of excess deaths by suicide (ED = O in a certain period – E in the same period) with the corresponding 95% CI [6] was selected as well. These analyses were done for both the total number of suicides and specific numbers of suicides (e.g., gender-specific suicide data) [6].

In order to answer the question whether the ESMR for the lockdown period in Germany in 2020–2021 was significantly different from the ESMR for the non-lockdown periods (in Germany in 2020 (period between the first and second lockdown) and in 2021 (post-lockdown period)), respectively the 95% CI for these rates were compared. If there was an overlap of the CI the ESMR difference failed to be statistically significant.

Moreover, the weekly registered and predicted number of suicides had been analyzed for the years 2020 and 2021 separately. Based on these calculations, a multi-factor analysis of variance (MANOVA) had been conducted. The dependent variable was the weekly absolute number of suicides and the independent variables were “year” (2020/2021) and “lockdown” (yes/no). Thus, the questions could be addressed whether there were significant annual changes of the number of suicides in the selected two-year period (2020–2021) and whether there were significant overall differences between lockdown and non-lockdown periods in this time interval. In addition, a possible interaction of the factors “year” and “lockdown” was examined. All these analyses were done for both the registered and predicted number of suicides.

In order to answer the question of whether the percentage of more lethal suicide methods (e.g., hanging) among all suicides in the total population during the lockdown period in 2020–2021 was significantly different from that in the corresponding non-lockdown periods (2020: weeks 20–50; 2021: weeks 9–52), chi-square tests for two-by-two cross tables were calculated. The corresponding rows represented the suicide methods (e.g., poisoning and all other suicide methods) and the columns the period (lockdown versus non-lockdown period).

SPSS version 29.0 was used for the statistical analyses. The significance level was α = 0.05. All statistical tests were two-tailed.

## Results

### Excess suicide mortality rates during the COVID-19 period in 2020–2021

During the COVID-19 period in 2020 (weeks 13–52) and 2021 (weeks 1–52), 16,256 individuals died from suicide in Germany and 15,998 individuals were expected to die based on findings for this period during the years 2010–2019. The total excess suicide mortality rate (ESMR) was 1.0161 (95% CI: 1.0005; 1.0317). So, the registered suicide mortality was significantly higher than expected. In line with this finding, the excess deaths from suicide (258; 95% CI: 8.10; 507.90) were significantly higher than expected.

### Excess suicide mortality rates during the lockdown periods in 2020–2021

In 2020–2021, 2,837 persons died by suicides during the lockdown periods in Germany. The total ESMR was 0.9477 (95% CI: 0.9128; 0.9825; see Table 1), indicating a significantly lower registered suicide mortality as compared with the expected one. In line with this result, the excess deaths from suicide (−156.64; 95% CI: −261.04; −52.24; see Table 1) were significantly lower than 0. The results of subgroup analyses are shown in Table 1, too. Registered suicide mortality was found to be significantly lower than expected in the case of following subgroups: men; individuals aged <25 years; individuals with an age of 45–64 years and men aged 45–64 years.

**Table 1:** Excess suicide mortality rates during the first and second lockdown in Germany in 2020-2021.

Group	Expected suicide rate	Ob-served suicide rate	Expected number of suicides (E)	Observed number of suicides (O)	ESMR (95% CI)	Excess number of suicides (95% CI)
**Total** ^1^	3.5984	3.4102	2993.64	2837	0.9477 (0.9128: 0.9825)	−156.64 (−261.04; −52.24)
**Men** ^1^	5.4751	5.1723	2247.26	2123	0.9447 (0.9045; 0.9849)	−124.26 (−214.57; −33.95)
**Women** ^1^	1.7613	1.6940	742.34	714	0.9618 (0.8913; 1.0324)	−28.34 (−80.71; 24.03)
**Age groups**	---	---	---	---	---	---
**≤24 years men and women** ^1^	0.7887	0.6473	157.18	129	0.8207 (0.6791; 0.9623)	−28.18 (−50.44; −5.92)
**25-44 years men and women** ^1^	2.5730	2.6409	536.81	551	1.0264 (0.9407; 1.1121)	14.19 (−31.82; 60.20)
**45-64 years men and women** ^1^	4.4232	4.0661	1063.90	978	0.9193 (0.8616; 0.9769)	−85.90 (−147.20; −24.60)
**≥65 years men and women** ^1^	6.6930	6.4261	1228.00	1179	0.9601 (0.9053; 1.0149)	−49.00 (−116.30; 18.30)
**45-64 years men** ^1^	6.5467	5.9763	786.52	718	0.9129 (0.8461; 0.9797)	−68.52 (−121.04; −16.00)
**45-64 years women** ^1^	2.3027	2.1598	277.22	260	0.9379 (0.8239; 1.0519)	−17.22 (−48.82; 14.38)
**≥65 years men** ^1^	11.2946	10.6302	910.65	857	0.9411 (0.8781; 1.0041)	−53.65 (−111.03; 3.73)
**≥65 years women** ^1^	3.0739	3.1308	316.13	322	1.0186 (0.9073; 1.1298)	5.87 (−29.30; 41.04)

**Notes:** CI = confidence interval; ESMR = excess suicide mortality rate. For four groups (≤24 years men; ≤ 24 years women; 25–44 years men and 25–44 years women) the calculation of the expected number of suicides and the expected suicide rate was not applicable because of missing values regarding subgroup-specific suicide data for the period 2011–2013 due to restrictions of the German data protection law (for the first subperiod of the second lockdown period the corresponding numbers were too small to prevent interested parties from the identification of single suicide cases). The observed number of suicides was for these groups as follows: ≤ 24 years men: 96; ≤ 24 years women: 33; 25–44 years men: 452; 25–44 years women: 99. The observed suicide rates were as follows: ≤ 24 years men: 0.9328; ≤ 24 years women: 0.3423; 25–44 years men: 4.2331; 25–44 years women: 0.9720.

^1^Prediction from the results from three regression analyses. The first one was based on annual suicide rates for the period of the first lockdown between 2010 and 2019. The second one was based on annual suicide rates for the subperiod week 51–53 (regarding the second lockdown period in 2020) between 2010 and 2019, the third one on annual suicide rates for the subperiod week 1–8 (regarding the second lockdown period in 2021) between the same years.

### Excess suicide mortality rates during the non-lockdown periods in 2020/2021

In 2020–2021, 13,419 individuals died from suicide during the non-lockdown periods in Germany (period between the first and second lockdown in 2020 (week 20–50) + post-lockdown period in 2021 (week 9–52)) whereas 12961 individuals (exact predicted number: 12961.34) were expected to die. The corresponding suicides rates were 16.13/100,000 and 15.58/100,000, respectively. Total ESMR was 1.0353 (95% CI: 1.0178; 1.0528); thus, the registered suicide mortality was significantly higher than expected. The excess number of deaths from suicide (457.66; 95% CI: 230.61; 684.71) was also significantly higher than expected.

### Suicide mortality comparisons between the lockdown and non-lockdown periods in 2020–2021

The 95% CI for both ESMR and excess deaths from suicide during the lockdown and non-lockdown periods for the total population in Germany in 2020–2021 were characterized by the lack of an overlap. Thus, they significantly differed from each other.

The weekly registered absolute numbers of suicides for the non-lockdown periods (2020: mean = 178.65; standard deviation (s.d.) = 15.09; 2021: mean = 179.59; s.d. = 15.26) were found to be markedly higher than the corresponding number of suicides for the lockdown periods (2020: mean = 169.33; s.d. = 16.17; 2021: mean = 164.13; s.d. = 13.73). A MANOVA revealed a significant difference between lockdown and non-lockdown periods (factor “lockdown”: F(1,100) = 9.47; p = 0.003) whereas annual differences failed to be statistically significant (factor “year”: F(1,100) = 0.28; p = 0.60). The interaction of the factors “lockdown” and “year” failed to be significant, too (F(1,100) = 0.58; p = 0.45).

Regarding the predicted absolute numbers of suicides for the non-lockdown periods (2020: mean = 176.63; s.d. = 13.67; 2021: mean = 176.07; s.d. = 15.04) they were similar to the corresponding numbers for the lockdown periods (2020: mean = 179.33; s.d. = 12.96; 2021: mean = 183.38; s.d. = 15.03). According to a MANOVA there was neither a significant main effect of the factor “year” (F(1,100) = 0.21; p = 0.65) nor a significant main effect of the factor “lockdown” (F(1,100) = 1.73; p = 0.19). The interaction of the factors “lockdown” and “year” failed to be statistically significant, too (F(1,100) = 0.37; p = 0.55).

The weekly absolute numbers of suicides for the total population in Germany in the period 2020–2021 are summarized in S1 Table.

### Suicide methods in 2020–2021 during the lockdown periods compared to non-lockdown periods in Germany

The frequencies for suicide methods during the lockdown periods in Germany in 2020–2021 as compared to the corresponding non-lockdown periods are summarized in Table 2. Overall, the corresponding differences failed to be significant. The same was true for the subgroups of men and women.

**Table 2:** Differences between lockdown and non-lockdown phases in 2020 and 2021 in Germany regarding the percentages of selected suicide methods.

	Lockdown periods (n(%))	Non-lock-down periods (n(%))	χ^2^	p value
**All**	(N = 2837)	(N = 13419)	---	---
**Self-Poisoning**	500 (17.62%)	2404 (17.91%)	0.13	0.71
**Hanging**	1249 (44.03%)	5966 (44.46%)	0.18	0.67
**Drowning**	64 (2.26%)	280 (2.09%)	0.32	0.57
**Firearms**	204 (7.19%)	942 (7.02%)	0.10	0.75
**Jumping**	297 (10.47%)	1384 (10.31%)	0.06	0.81
**Sharp objects**	142 (5.01%)	575 (4.28%)	2.88	0.09^+^
**Moving objects**	166 (5.85%)	873 (6.51%)	1.68	0.20
**Other suicide methods**	215 (7.58%)	995 (7.41%)	0.09	0.76
**Males**	(n = 2123)	(n = 9961)	---	---
**Self-Poisoning**	275 (12.95%)	1317 (13.22%)	0.11	0.74
**Hanging**	1027 (48.37%)	4892 (49.11%)	0.38	0.54
**Jumping**	211 (9.94%)	929 (9.33%)	0.77	0.38
**Sharp objects**	114 (5.37%)	451 (4.53%)	2.78	0.095^+^
**Moving objects**	127 (5.98%)	672 (6.75%)	1.66	0.20
**Other suicide methods**	146 (6.88%)	639 (6.42%)	0.62	0.43
**Females**	(n = 714)	(n = 3458)	---	---
**Self-Poisoning**	225 (31.51%)	1087 (31.43%)	0.002	0.97
**Hanging**	222 (31.09%)	1074 (31.06%)	0.0003	0.99
**Jumping**	86 (12.04%)	455 (13.16%)	0.65	0.42
**Sharp objects**	28 (3.92%)	124 (3.59%)	0.19	0.66
**Moving objects**	39 (5.46%)	201 (5.81%)	0.13	0.71
**Other suicide methods**	69 (9.66%)	356 (10.29%)	0.26	0.61

**Notes:** N = sample size; n = group size. The calculation of the number of the suicide methods “drowning” and “firearms” in the lockdown periods for men and women separately was not applicable (due to small numbers and correspondent restrictions of the German data protection law). For drowning the corresponding numbers in the non-lockdown periods were as follows: men: 146 (1.47%); women: 134 (3.88%). For firearms the corresponding numbers in the non-lockdown periods were as follows: men: 915 (9.19%); women: 27 (0.78%).

+p < 0.10.

The results were based on chi-square (χ^2^) tests for two-by-two tables.

Suicide methods were defined as follows according to ICD-10 codes X60-X84: Poisoning (X60-X69), hanging (X70), drowning (X71), firearms (X72-X75), sharp objects (X78), jumping (X80), moving objects (X81, X82), other suicide methods (X76, X77, X79, X83, X84).

## Discussion

In the present study, we analyzed suicide data of the years 2020 and 2021 from Germany during the lockdown and non-lockdown periods in the context of the COVID-19 pandemic compared to a ten-year baseline. Suicide rates in Germany were higher than expected during the COVID-19 pandemic in the years 2020 and 2021. We found that during the two lockdowns in these years, the suicide mortality was lower than expected. On the contrary, the non-lockdown phases showed an increase in suicide mortality. It also became apparent that less suicides were registered during lockdown compared to non-lockdown phases. We could not detect a shift in suicide methods when looking at lockdown vs. non-lockdown phases.

With the availability of the 2021 suicide numbers, more suicide data could be analyzed than in the previous analysis [6] and thus power was increased in the area of statistically small suicide numbers during the different phases, especially during the shorter lockdown intervals. The current results enforce the findings from the first study in that both lockdowns show decreasing suicide numbers and hint towards a suicide preventive effect of lockdowns. While it remained unclear in our first study whether the decreased suicide mortality during the first lockdown represented a general suicide preventive effect or rather a state of shock in view of the global novelty of the situation, the analyses of this study indicate that the latter does not fully explain the decreasing suicide mortality.

Mechanisms behind the decreased suicide rates during lockdowns could be increased social control, e.g., less opportunities for dying by suicide in homes as well as difficulty of access to different suicide methods. Also, it might be possible that the “pulling together effect”, sometimes also called “honeymoon phase” has played a role. This effect was first described by Durkheim, who postulated that for the example of war, people pull together during phases of collective adversity and suicide rates sink because of this increased social integration [12]. This phenomenon has also been observed after natural disasters [13]. It has been taken up as a consideration that “pulling together” has also been in effect concerning the COVID-19 pandemic as an interpretation of the finding that suicide rates in some countries were decreasing in the beginning of the pandemic and later increased [1,8,14–16]. Reger proposes that the shared experience of the COVID-19 pandemic might have given another view on health as a more valuable asset and thereby reduce suicides [1]. Also, since the measures to curb the spread of the virus required a joint action from the community (e.g., reducing physical contacts), this might have contributed to the enhanced feeling of social connection. It was also pointed out, that this social cohesion showed in the public concern for at-risk groups and the question of how to maintain social inclusion despite the demanded physical distance [15,17].

On the other hand, higher suicide mortality during the non-lockdown phases, i.e., after the first and after the second lockdown in 2020 and 2021 is concerning. Reasons for this increase could be a kind of “postponement effect”: Suicidality might be present during lockdown phases in patients, but the realization of suicides is hindered due to the lockdown restrictions. With the loosening of the restrictions, suicide methods might be more easily accessible again and thus lead to more suicides. This is in line with the finding in our study which showed that there was no shift in suicide methods when comparing lockdown and non-lockdown phases. In order to better understand these dynamics, a representative assessment of suicide attempts would be required.

On a broader scale, the rise of suicide mortality since the start of the COVID-19 pandemic is an alarming finding of our study. This is in line with the increased suicide mortality of the year 2022 in Germany, where we saw a rise of about 10% of the absolute number of suicides compared to 2021 and thus the highest increase since the year 1980 [18]. After international studies showed unchanged or decreased suicide mortality in the first months of the COVID-19 pandemic [7,8], some have reported increasing suicide numbers after the first months [16,19,20]. This is an indicator that the far-reaching and delayed consequences of the COVID-19 pandemic and its measures to curb the spread of the virus have to be acknowledged. Possible reasons for this development could be the long-term worsening of the course of mental disorders, especially depression [21], and thereby increasing suicidal behavior.

In order to better understand the mechanisms during the course of the COVID-19 pandemic concerning suicide mortality, more research is needed. There are various factors known to be associated with suicidality such as, e.g., economic recession, especially unemployment, or quality of care [22–25], which might have been altered by the pandemic. In the scope of this study, we decided to not include additional factors from other data sources that might influence suicide mortality, since a selection of single factors was difficult and not the focus of this analysis. Including single or several of the above named factors might be an approach in future studies.

One limitation of our study is that our findings are not generalizable for the whole time-span of the COVID-19 pandemic. This is due to the restricted data availability: Since weekly suicide data were only available for the years 2020 and 2021 at the time of the analyses, we did not have complete data for the whole time span of the COVID-19 pandemic. The official end of the COVID-19 pandemic was declared in May 2023 [26], thus data of 2022 and 2023 would need to be included in order to describe the whole time-span of the COVID-19 pandemic.

Furthermore, in our direct comparison of the suicide mortality of lockdown and non-lockdown phases, we could not control for seasonal variations due to methodological reasons.

It can be concluded that lockdown and non-lockdown phases in Germany during the early phase of the COVID-19 pandemic (2020−2021) had differential effects on suicide rates: significantly lower suicide rates than expected for the lockdown periods and significantly higher suicide rates than expected for the non-lockdown periods. These findings suggest a “postponement effect” in that the loosening of the lockdown restrictions makes suicide methods more easily accessible and more suicides than expected are present in the following non-lockdown periods. It will yet have to be examined whether these findings can be replicated for the whole COVID-19 time period.

## Supporting information

S1 Table Registered and expected absolute numbers of suicides per week in 2020–2021 in Germany. (DOCX)
