# Supplementary material for: Suicide rates in Germany in lockdown and non-lockdown phases during the COVID-19 pandemic in 2020/2021
Source: PLoS One. 2025 Sep 8;20(9):e0331784. doi: 10.1371/journal.pone.0331784 (PMC12416668; doi:10.1371/journal.pone.0331784)
Supplement: S1 Table — (DOCX) [file pone.0331784.s001.docx]

**Table S1.** **Registered and expected absolute numbers of suicides per week in 2020-2021 in Germany**

| **Week** | **Registered number of suicides** | **Expected number of suicides** | **Difference** | **p value** |
| --- | --- | --- | --- | --- |
| **2020** | | | | |
| **1** | 184 | 204.49 | -20.49 | 0.16 |
| **2** | 196 | 194.69 | 1.31 | 0.94 |
| **3** | 169 | 176.64 | -7.64 | 0.54 |
| **4** | 160 | 168.33 | -8.33 | 0.53 |
| **5** | 190 | 182.90 | 7.10 | 0.60 |
| **6** | 181 | 165.54 | 15.46 | 0.24 |
| **7** | 165 | 190.59 | -25.59 | 0.06^+^ |
| **8** | 187 | 170.51 | 16.49 | 0.22 |
| **9** | 202 | 175.95 | 26.05 | 0.048*^a^ |
| **10** | 161 | 203.12 | -42.12 | 0.003**^a^ |
| **11** | 196 | 169.84 | 26.16 | 0.044*^a^ |
| **12** | 174 | 166.08 | 7.92 | 0.53 |
| **13** | 162 | 182.72 | -20.72 | 0.12 |
| **14** | 179 | 198.15 | -19.15 | 0.17 |
| **15** | 153 | 188.87 | -35.87 | 0.008**^a^ |
| **16** | 174 | 177.51 | -3.51 | 0.76 |
| **17** | 164 | 176.44 | -12.44 | 0.36 |
| **18** | 175 | 174 | 1 | 0.94 |
| **19** | 189 | 193.33 | -4.33 | 0.77 |
| **20** | 198 | 196.99 | 1.01 | 0.94 |
| **21** | 204 | 184.13 | 19.87 | 0.14 |
| **22** | 180 | 195.61 | -15.61 | 0.25 |
| **23** | 195 | 196.69 | -1.69 | 0.89 |
| **24** | 213 | 179.91 | 33.09 | 0.013*^a^ |
| **25** | 198 | 184.14 | 13.86 | 0.30 |
| **26** | 181 | 177.41 | 3.59 | 0.76 |
| **27** | 177 | 180.94 | -3.94 | 0.76 |
| **28** | 170 | 160.05 | 9.95 | 0.43 |
| **29** | 186 | 194.62 | -8.62 | 0.52 |
| **30** | 191 | 194.21 | -3.21 | 0.83 |
| **31** | 192 | 170.74 | 21.26 | 0.11 |
| **32** | 181 | 150.93 | 30.07 | 0.014^*a^ |
| **33** | 179 | 175.38 | 3.62 | 0.76 |
| **34** | 170 | 174.59 | -4.59 | 0.70 |
| **35** | 174 | 175.97 | -1.97 | 0.88 |
| **36** | 176 | 172.64 | 3.36 | 0.82 |
| **37** | 178 | 150.65 | 27.35 | 0.027*^a^ |
| **38** | 175 | 164.49 | 10.51 | 0.39 |
| **39** | 170 | 161.50 | 8.50 | 0.53 |
| **40** | 193 | 161.25 | 31.75 | 0.011*^a^ |
| **41** | 156 | 180.10 | -24.10 | 0.07^+^ |
| **42** | 156 | 182.04 | -26.04 | 0.052^+^ |
| **43** | 166 | 174.19 | -8.19 | 0.54 |
| **44** | 181 | 168.59 | 12.41 | 0.35 |
| **45** | 175 | 192.17 | -17.17 | 0.22 |
| **46** | 159 | 158.52 | 0.48 | 1 |
| **47** | 181 | 167.80 | 13.20 | 0.31 |
| **48** | 159 | 164 | -5 | 0.69 |
| **49** | 157 | 175.76 | -18.76 | 0.15 |
| **50** | 146 | 157.77 | -11.77 | 0.34 |
| **51** | 140 | 157.80 | -17.80 | 0.15 |
| **52** | 188 | 164.68 | 23.32 | 0.07^+^ |
| **2021** | | | | |
| **1** | 155 | 206.86 | -51.86 | **<0.001***** |
| **2** | 160 | 197.72 | -37.72 | 0.007**^a^ |
| **3** | 154 | 177.37 | -23.37 | 0.076^+a^ |
| **4** | 177 | 170.05 | 6.95 | 0.591 |
| **5** | 179 | 185.34 | -6.34 | 0.638 |
| **6** | 144 | 166.74 | -22.74 | 0.076^+a^ |
| **7** | 162 | 194.20 | -32.20 | 0.020*^a^ |
| **8** | 182 | 169.10 | 12.90 | 0.317 |
| **9** | 171 | 174.83 | -3.83 | 0.770 |
| **10** | 180 | 206.53 | -26.53 | 0.062^+a^ |
| **11** | 172 | 166.58 | 5.42 | 0.672 |
| **12** | 163 | 162.15 | .85 | 0.946 |
| **13** | 157 | 184.52 | -27.52 | 0.041*^a^ |
| **14** | 178 | 198.41 | -20.41 | 0.143 |
| **15** | 175 | 188.53 | -13.53 | 0.319 |
| **16** | 181 | 176.32 | 4.68 | 0.722 |
| **17** | 172 | 175.49 | -3.49 | 0.790 |
| **18** | 171 | 171.36 | -.36 | 0.978 |
| **19** | 174 | 194.37 | -20.37 | 0.140 |
| **20** | 165 | 200.43 | -35.43 | 0.011*^a^ |
| **21** | 190 | 185.44 | 4.56 | 0.735 |
| **22** | 182 | 199.45 | -17.45 | 0.212 |
| **23** | 199 | 197.70 | 1.30 | 0.926 |
| **24** | 181 | 180.62 | .38 | 0.977 |
| **25** | 197 | 185.74 | 11.26 | 0.404 |
| **26** | 156 | 177.08 | -21.08 | 0.110 |
| **27** | 191 | 179.85 | 11.15 | 0.401 |
| **28** | 156 | 155.88 | .12 | 0.992 |
| **29** | 186 | 196.46 | -10.46 | 0.451 |
| **30** | 169 | 196.99 | -27.99 | 0.044*^a^ |
| **31** | 167 | 169.11 | -2.11 | 0.870 |
| **32** | 199 | 147.11 | 51.89 | **<0.001***** |
| **33** | 181 | 174.91 | 6.09 | 0.642 |
| **34** | 171 | 174.76 | -3.76 | 0.774 |
| **35** | 171 | 175.94 | -4.94 | 0.707 |
| **36** | 204 | 172.28 | 31.72 | 0.015*^a^ |
| **37** | 172 | 145.73 | 26.27 | 0.028*^a^ |
| **38** | 158 | 164.30 | -6.30 | 0.620 |
| **39** | 195 | 159.26 | 35.74 | 0.004**^a^ |
| **40** | 195 | 159.77 | 35.23 | 0.005**^a^ |
| **41** | 205 | 181.03 | 23.97 | 0.072^+a^ |
| **42** | 191 | 184.69 | 6.31 | 0.639 |
| **43** | 174 | 174.02 | -.02 | 0.999 |
| **44** | 191 | 168.91 | 22.09 | 0.086^+a^ |
| **45** | 191 | 194.46 | -3.46 | 0.802 |
| **46** | 166 | 156.04 | 9.96 | 0.421 |
| **47** | 207 | 167.38 | 39.62 | 0.002**^a^ |
| **48** | 161 | 164.34 | -3.34 | 0.793 |
| **49** | 212 | 177.65 | 34.35 | 0.009**^a^ |
| **50** | 195 | 157.05 | 37.95 | 0.002**^a^ |
| **51** | 174 | 159.53 | 14.47 | 0.248 |
| **52** | 156 | 164.60 | -8.60 | 0.499 |

n.s. = not significant; ^+^ p < 0.10; * p < 0.05; ** p < 0.01; *** p < 0.001.

^a^ The difference is not statistically significant after alpha-adjustment for multiple testing (α = 0.05/52 = 0.00096).

Significant findings after alpha-adjustment for multiple testing are in bold.

The expected values for the total sample were based on annual suicide rates for single weeks between 2010 and 2019.

In this context, the population figures used for the calculation of suicide rates were derived from end-period registry counts as given in a publicly accessible database (genesis) from the Federal Statistical Office of Germany (for details see: <https://www-genesis.destatis.de/genesis//online?operation=table&code=12411-0006&bypass=true&levelindex=0&>levelid=1658420460497#abreadcrumb).
